# Loss of *dpy-2* and* dpy-9* has stage-specific effects on DBL-1 pathway signaling

**DOI:** 10.17912/micropub.biology.000191

**Published:** 2019-12-09

**Authors:** Mohammed Farhan Lakdawala, Tina L. Gumienny

**Affiliations:** 1 Department of Biology, Texas Woman's University, Denton, TX, 76204-5799

**Figure 1:**
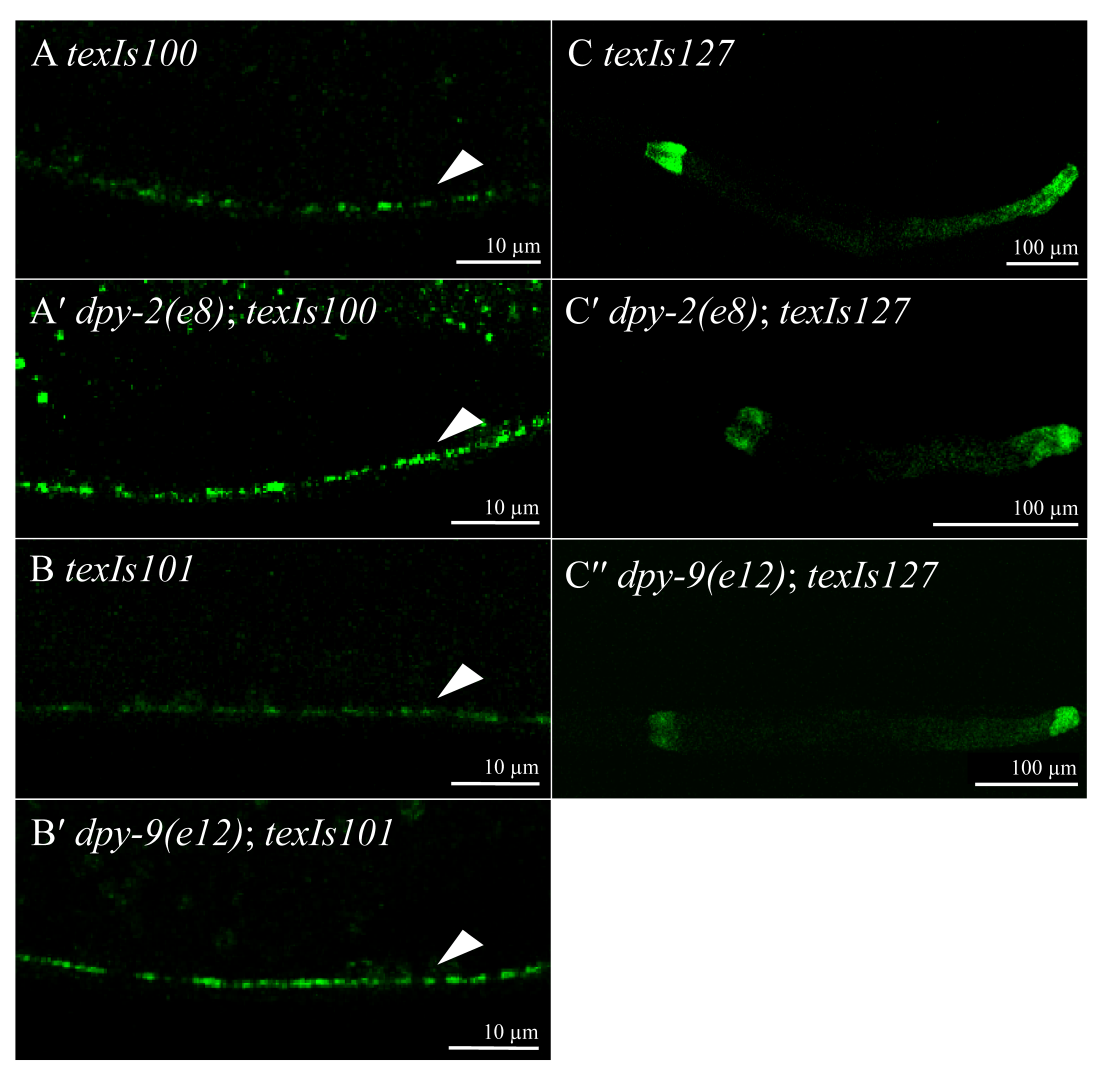
*dpy-2* or *dpy-9* loss-of-function mutations affect GFP::DBL-1 and DBL-1 pathway reporter fluorescence in L4 animals. Arrows point to GFP::DBL-1 fluorescent punctae in A and B. Representative images show that loss of *dpy-2* or *dpy-9* gene function is associated with increased GFP::DBL-1 fluorescence from *texIs100* or *texIs101* as shown in (A′) and (B′), respectively. *dpy-2(e8)* and *dpy-9(e12)*mutants also have reduced *spp-9p::gfp* reporter activity compared to control (C), as shown in (C′) and (C′′), respectively.

## Description

Loss of some cuticle collagens negatively affects DBL-1 pathway signaling in a stage-dependent manner (Lakdawala *et al.* 2019; Madaan *et al.* 2019). We previously observed that in one-day old adult animals, loss of *dpy-2* or *dpy-9* had no effect on GFP::DBL-1 expressed from the *dbl-1* promoter (Beifuss and Gumienny 2012; Lakdawala *et al.* 2019). We also observed that expression of *spp-9p::gfp*, a reporter that is negatively regulated by the DBL-1 pathway, was not affected in one-day old adult animals (Roberts *et al.* 2010; Lakdawala *et al.* 2019). Post-embryonic expression of *dpy-2* and *dpy-9* is highest in L2 and L3, but low in L4 and even lower in young adults (Gerstein *et al.* 2010). Because cuticle secreted in one stage creates the cuticle in the next stage, this is consistent with the observation that loss of *dpy-2* and *dpy-9* has no effect on DBL-1 signaling in the adult (Hall and Altun 2008; Lakdawala *et al.* 2019). However, the DPY-2 and DPY-9 expression patterns led us to ask if DBL-1 signaling is affected at L4 by loss of *dpy-2* or *dpy-9*. To our surprise, we found that *dpy-2(e8)* or *dpy-9(e12)* resulted in significant increases of GFP::DBL-1 fluorescence within DBL-1-secreting cells in L4 animals compared to control populations (Figure 1, Table 1). We also tested DBL-1 pathway reporter activity in these *dpy-2* and *dpy-9* mutants. Consistent with the increased GFP::DBL-1 fluorescence at L4, we observed significantly decreased fluorescence from the *spp-9p::gfp* reporter at L4 (Figure 1, Table 1). These results are consistent with DPY-2 and DPY-9 affecting DBL-1 signaling at the L4 stage but not at the adult stage. This suggests that these two collagens have a stage-specific effect on DBL-1 signaling, but this effect is normally inhibitory, as loss of *dpy-2* or *dpy-9* increased GFP::DBL-1 fluorescence and decreased *spp-9p*::GFP fluorescence.

**Table:** 

Table 1: Effects of *dpy-2* and *dpy-9* gene mutations on GFP::DBL-1 and DBL-1 pathway reporter *spp-9p*::GFP fluorescence						
Gene	Genotype	GFP::DBL-1 fluorescence % control ± 95% CI	P value	Genotype	*spp-9p*::GFP % control ± 95% CI	P value
Animals at L4 stage						
control	*texIs100*	100±29.58	–	*texIs127*	100±7.94	–
control	*texIs101*	100±54.28	–	–	–	–
*dpy-2*	*dpy-2; texIs100*	155.47±55.58	0.0263	*dpy-2; texIs127*	80.26±10.62	0.0009
*dpy-9*	*dpy-9; texIs101*	212.94±98.06	0.0009	*dpy-9; texIs127*	84.37±9.56	0.0028
Animals at adult stage (data from (Lakdawala *et al.* 2019))						
control	*texIs100*	100±15.57	–	*texIs127*	100±11.47	–
control	*texIs101*	100±25.95	–	–	–	–
*dpy-2*	*dpy-2; texIs100*	115±52.15	0.5080	*dpy-2; texIs127*	107.04±12.20	0.2344
*dpy-9*	*dpy-9; texIs101*	95.02±29.01	0.7248	*dpy-9; texIs127*	100.29±10.24	0.9533

## Methods

Nematode maintenance and imaging All the strains were maintained at 20°C on EZ media (Madhu et al. 2019). L4 animals were anesthetized using 1 mM levamisole hydrochloride (Sigma, St. Louis, MO) and imaged on a Nikon A1 confocal system (Nikon Instruments, Melville, NY). GFP::DBL-1 fluorescence was captured using a 60X objective and *spp-9p::gfp* fluorescence was captured using a 10X objective. The imaging conditions were optimized and kept constant between control and experimental samples. Nikon NIS Elements AR-5.02 software was used to quantify fluorescence intensities. Statistical analyses were performed using the unpaired t-test to compare control and experimental sample means. ‘‘% control ± 95% CI” is the ratio of the indicated strain mean to the control strain mean ± 95% confidence interval. n=10 for each strain imaged for the GFP::DBL-1 experiment, and n=15 for each strain imaged for the *spp-9p*::GFP experiment.

## Reagents

**Strains**

Strains used in this study are:

TLG182 *texIs100* *[dbl-1::dbl-1:gfp; ttx-3p*::*rfp]* IV

TLG205 *texIs101 [dbl-1*::*dbl-1*:*gfp*; *ttx-3p*::*rfp]* V

TLG697 *texIs127 [spp-9p::gfp] X*

TLG701 *dpy-2(e8); texIs100*

TLG702 *dpy-9(e12); texIs101*

TLG725 *dpy-2(e8); texIs127*

TLG724 *dpy-9(e12); texIs127*

Strains are available upon request.
